# Severe infection increases cardiovascular risk among HIV-infected individuals

**DOI:** 10.1186/s12879-019-3894-6

**Published:** 2019-04-11

**Authors:** Emersom Cicilini Mesquita, Lara Esteves Coelho, Rodrigo Teixeira Amancio, Valdilea Veloso, Beatriz Grinsztejn, Paula Luz, Fernando Augusto Bozza

**Affiliations:** 1 Laboratório de Pesquisa Clínica em Medicina Intensiva, Instituto Nacional de Infectologia Evandro Chagas (INI), Fundação Oswaldo Cruz (FIOCRUZ), Rio de Janeiro, Brazil; 2 Laboratório de HIV, Instituto de Nacional de Infectologia Evandro Chagas (INI), Fundação Oswaldo Cruz (FIOCRUZ), Rio de Janeiro, Brazil; 3 Instituto D’Or de Pesquisa e Ensino (IDOR), Rio de Janeiro, Brazil

**Keywords:** Cardiovascular disease, Risk factor, HIV, AIDS, Severe infection, cART, CD4+

## Abstract

**Background:**

The identification and management of cardiovascular risk factors became a major clinical issue among HIV-infected individuals in the post-cART era. As in the past decades the link between acute infections and cardiovascular diseases became clear in the general population, we sorted to investigate the role of severe infections on incident cardiovascular diseases (CVDs) among HIV-infected individuals.

**Methods:**

HIV-infected individuals aged ≥18 years, with no history of CVD were followed from January 2000 to December 2013 until the occurrence of the first CVD event, death or end of study, whichever occurred first. To explore the effect of severe infections on the incidence of CVD we used extended Cox regression models and stratified post-hospitalization follow-up time into three periods: < 3 months, 3–12 months and > 12 months post discharge.

**Results:**

One hundred-eighty four persons from 3384 HIV-infected individuals developed incident CVD events during the follow-up (incidence rate = 11.10/1000 PY (95%CI: 9.60–12.82)). Risk of an incident CVD was 4-fold higher at < 3 months post-hospitalization for severe infections (adjusted hazard ratio [aHR], 4.52; 95% confidence interval [CI] 2.46–8.30), after adjusting for sociodemographic and clinical factors as well as comorbidities. This risk remained significant up to one year (3–12 months post hospital discharge aHR 2.39, 95% CI 1.30–4.38). Additionally, non-white race/ethnicity (aHR 1.49, 95% CI 1.10–2.02), age ≥ 60 years (aHR 2.01, 95% CI 1.01–3.97) and hypertension (aHR 1.90, 95% CI 1.38–2.60) were associated with an increased risk of CVD events. High CD4 (≥ 500 cells/mm^3^: aHR 0.41, 95% CI 0.27–0.62) and cART use (aHR 0.21, 95% CI 0.14–0.31) reduced the risk of CVD events.

**Conclusions:**

We provide evidence for a time-dependent association between severe infection and incident cardiovascular disease in HIV-infected individuals. cART use, and high CD4 count were significantly associated with reduced hazards of CVD.

**Electronic supplementary material:**

The online version of this article (10.1186/s12879-019-3894-6) contains supplementary material, which is available to authorized users.

## Background

With introduction and widespread use of combined antiretroviral therapy (cART) a marked shift in causes of hospitalizations and deaths have been observed among HIV-infected individuals, with a relative increase in non-communicable diseases (NCD) and non-AIDS-defining infections [1–3]. Among NCD, cardiovascular diseases (CVD) represent a major challenge. HIV-infected individuals have a higher risk for CVD such as myocardial infarction (MI), stroke, heart failure, and sudden cardiac death [4–7]. Although a higher burden of traditional CVD risk factors have been reported in HIV-infected individuals [8, 9], an elevated risk remains even after adjusting for these factors [10, 11]. Additionally, chronic exposure to cART [12, 13], HIV infection itself, and HIV-specific clinical parameters, such as detectable HIV-1 RNA, and nadir CD4+ count ≤50 cells/mm^3^, may also contribute to this increased CVD risk [14, 15].

As AIDS became a chronic condition [16], non-AIDS, life-threatening bacterial infection and sepsis grew as cause of hospitalization in this population [17]. In the intensive care scenario, sepsis is the major determinant of outcome in HIV-infected individuals [18, 19]. Interestingly, despite the presence of severe immunosuppression, a robust innate immune response is present and associated with hospital mortality [20]. In this study, we hypothesized that HIV-infected individuals who were hospitalized for severe infections would have an increased CVD risk compared to HIV-infected individuals without hospitalization for severe infections. Identifying specific CVD risk in this population is critical for guiding targeted CVD prevention and treatment.

## Methods

### Data sources and study population

The Instituto Nacional de Infectologia Evandro Chagas (INI, formerly known as Instituto de Pesquisa Clínica Evandro Chagas/IPEC) provides primary and specialized health care for HIV-infected individuals in Rio de Janeiro State, Brazil. It comprises an outpatient clinic, emergency department and inpatient care, including an intensive care unit. All health care services provided by INI are funded by Brazilian’s public health system (known as Sistema Único de Saúde, SUS). A longitudinal observational cohort including socio-demographic, laboratory and clinical data has been maintained at INI since 1998. Data of patients receiving primary care at INI is abstracted from medical records and is updated periodically by trained staff. Cohort procedures have been described and results published [3, 21, 22].

For this study, from January 12,000 until December 312,013, we included HIV-infected individuals aged 18 years old or older, who were followed in the cohort for at least 60 days. The start of the follow-up was defined as the first medical appointment at INI. The outcome of interest was the incidence of the first CVD during follow-up such that for those who developed incident CVD, follow-up ended on the day of the diagnosis. For those not presenting any CVD, follow-up ended on the death date, last clinical visit, last blood drawn or study closure (December 312,013), whichever occurred first. Patients with history of cardiovascular disease (CVD) prior to cohort enrollment (*n* = 59 patients) were excluded from the study population. Lost to follow-up (LTFU) was defined for patients known not to be deceased as not having a clinical visit (medical visit, CD4, HIV viral load) after January 12,013. Patients LTFU contributed to the follow-up time up to the date of last clinical visit.

### Study definitions

We defined severe infections as any hospitalization related to any acute infection diagnosis (based on the discharge summary), which included ICD-10 codes for bacterial, viral, fungal or parasites infections (Additional file 1: Figure S1).

Cardiovascular diseases were defined as: coronary heart disease (CHD), stroke, peripheral arterial disease, deep venous thrombosis (DVT), pulmonary embolism, disorders of the heart muscle – cardiomyopathies – and disorders of the rhythm [23] (Additional file 2: Table S1). In cases when the CVD occurred during the hospitalization for infections, this hospitalization counted as an outcome (CVD) but not as an independent variable (severe infection). The following co-morbidities were defined up to patient’s end of follow-up. Hepatitis B and C co-infections were defined as having a positive HBsAg antigen and by the presence of anti-HCV antibodies, respectively. Arterial hypertension was defined as use of antihypertensive medication, systolic blood pressure > 140 mmHg, or diastolic blood pressure > 90 mmHg [15, 24] prior to the end of follow-up. Diabetes was defined as use of diabetes mellitus pharmacological treatment or a fasting glucose level ≥ 126 mg/dL or a hemoglobin A1c value of 6.5 or greater [15, 25, 26] prior to end of follow-up. Dyslipidemia was defined by the use of lipid-lowering therapy, low-density lipoprotein (LDL) cholesterol > 159 mg/dL, high-density lipoprotein (HDL) cholesterol < 40 mg/dL, total cholesterol > 239 mg/dL, or triglycerides > 199 mg/dL [15, 27] prior to the end of follow-up. cART use was defined for those who had 60 days or more of exposure to at least three antiretroviral drugs: two nucleoside reverse transcriptase inhibitors and either one protease inhibitor, one non-nucleoside reverse transcriptase inhibitor or one integrase inhibitor. ART adherence information was not available such that ART use was presumed for the entire follow-up time for those meeting the criteria described above. Tobacco ever use and cocaine ever use (comprises inhaled, smoked and injection use) were defined based on self-report.

### Statistical methods

Incidence rates of cardiovascular disease were calculated by dividing the number of events by the person-years at risk. Rates were calculated per 1000 person-years (PY) with respective 95% confidence interval using Poisson regression model. To explore the effect of severe infections on the incidence of CVD we used extended Cox regression models. We stratified a patient’s post-infection follow-up time into three periods as follows: < 3 months post-hospitalization, 3–12 months post-hospitalization and more than a year post-hospitalization. Descriptive statistics for demographic and clinical variables were compared using Kruskal-Wallis test for continuous variables and chi-squared for categorical variables. CD4 nadir was defined as the lowest CD4 count value available for each patient. Last CD4 count, last CD4/CD8 ratio and last HIV-RNA were defined as the last results within the patient’s final year of follow-up. Kaplan-Meier survival curves were plotted to show how the survival experience varied among groups. Beyond severe infections, other independent variables explored through unadjusted Cox regression models were age (as a time dependent variable), sex, race, HIV exposure category, educational level, hepatitis B and C co-infections, tobacco use, cocaine use, >arterial hypertension, diabetes, and dyslipidemia. The initial adjusted models included all variables explored in the unadjusted models that presented a *p*-value less than 0.20. The final model was achieved through backwards elimination until only significant variables, at the p-value of < 0.05, were present. Random imputation for missing values in viral loads, CD4 counts, and CD4/CD8 ratio was performed. Unadjusted and adjusted models without imputations for missing values were also fitted (Additional file 3: Table S2). R (version 3.0.3), library Survival were used for the analyses.

## Results

**Fig. 1:**
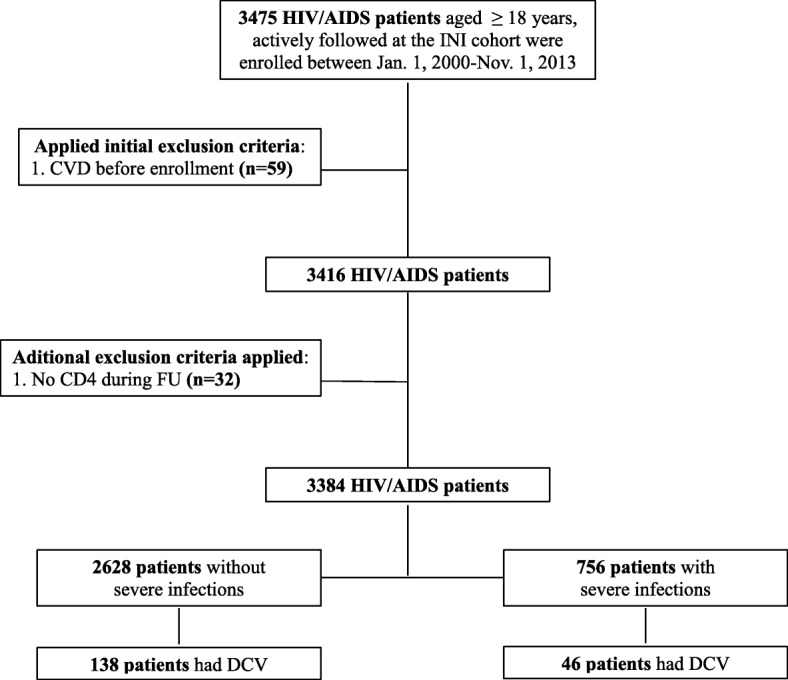
Study Participants Flowchart

Overall, 3475 HIV-infected individuals aged 18 years or more enrolled the INI cohort between January 12,000 and December 312,013. After excluding 59 HIV-infected individuals with past history of CVD and 32 who had no CD4+ cell count during follow-up, 3384 HIV-infected individuals were included in the present study (accounting for a total follow-up of 16,584 PY), with a median follow-up time of 4.4 years (interquartile range (IQR) 1.9–7.3 years). During the study period, 184 persons had CVD yielding an incidence rate of 11.10/1000 PY (95%CI: 9.60–12.82), and 178 (5.3%) individuals were lost to follow-up, yielding a rate of lost to follow-up of 10.73/1000 PY (95% CI, 9.27–12.43) (Fig. 1). A complete list of ICD-10 codes for CVD included in this analysis is provided in the supplementary material (Additional file 2: Table S1).

At baseline, median age was 40.5 years (IQR, 33.1–48.4), 68.3% were male, 50.7% non-white, and 52% had more than 9 years of schooling (Table 1). Tobacco and cocaine use were reported in 65.8 and 7.9%, respectively. The median nadir CD4+ count was 200 (IQR 71–325) cells/mm^3^ and 86.4% used cART for at least 60 days before end of follow-up. Median time of cART use was 4.5 years (IQR 2.1–8.0). Among those who did not have a CVD, median time was 4.5 years (IQR 2.2–8.0), and among those who had a CVD, median time was 3.4 years (IQR 1.3–6.8). Sixty-five percent of participants had a viral load less than 400 copies/mL in the year prior to their event/end of follow-up. Traditional CVD risk factors such as dyslipidemia, hypertension and diabetes mellitus were present in 46.3, 20.9 and 10% of all HIV-infected individuals, respectively, and were significantly more prevalent among participants with incident CVD.

**Table 1:** Characteristics of the study population

	No CVD	Had CVD	All patients	crude HR
	(*N* = 3200;16,029.2 PY)	(*N* = 184;554.4PY)	(*N* = 3384; 16,583.7PY)	(95% CI)
Median FU in years (IQR)	4.5 (2,7.3)	2 (0.6,4.3)	4.4 (1.9,7.3)	
Sex				
Male	2180 (68.1)	130 (70.7)	2310 (68.3)	ref.
Female	1018 (31.8)	56 (29.9)	1074 (31.7)	0.80 (0.58, 1.10)
Age in years at end of FU				
median (IQR)	40.2 (32.9,48.1)	46.6 (37.8,53.5)	40.5 (33.1,48.4)	
≤ 30 (%)	409 (12.8)	16 (8.7)	425 (12.6)	ref.
31–45 (%)	1692 (52.9)	64 (34.8)	1756 (51.9)	0.53 (0.31, 0.89)
46–59 (%)	958 (29.9)	88 (46.7)	1044 (30.9)	0.97 (0.58, 1.61)
≥ 60 (%)	141 (4.4)	18 (9.8)	159 (4.7)	1.34 (0.69, 2.60)
Race/ethnicity				
white (%)	1585 (49.5)	83 (45.1)	1668 (49.3)	ref.
non-white (%)	1615 (50.5)	101 (54.9)	1716 (50.7)	1.56 (1.16, 2.09)
Educational level				
up to 9 years (%)	1518 (47.4)	107 (58.2)	1625 (48)	ref.
more than 9 years (%)	1682 (52.6)	77 (41.8)	1759 (52)	0.67 (0.50, 0.90)
CD4 nadir (cells/mm^3^)				
median(IQR)	204 (71.8326.2)	140 (53,306)	200 (71,325)	1.03 (0.93, 1.13)
> 350 (%)	677 (21.2)	33 (17.9)	710 (21)	–
201–350 (%)	939 (29.3)	40 (21.7)	979 (28.9)	–
51–200 (%)	960 (30)	68 (37)	1028 (30.4)	–
≤ 50 (%)	624 (19.5)	43 (23.4)	667 (19.7)	–
Last HIV RNAª (copies/mL)				
< 400 (%)	2116 (66.1)	108 (58.7)	2224 (65.7)	ref.
≥ 400 (%)	746 (23.3)	62 (33.7)	808 (23.9)	2.40 (1.75, 3.31)
missing (%)	338 (10.6)	14 (7.6)	352 (10.4)	–
Last CD4ª (cells/mm^3^)				
median (IQR)	544.5 (341,741.2)	397 (222.5645.5)	537 (330.5739)	
< 350 (%)	765 (23.9)	80 (43.5)	845 (25)	ref.
350–499 (%)	540 (16.9)	28 (15.2)	568 (16.8)	0.40 (0.26, 0.63)
≥ 500 (%)	1663 (52)	71 (38.6)	1734 (51.2)	0.27 (0.19, 0.37)
missing (%)	232 (7.2)	5 (2.7)	237 (7)	–
Last CD4:CD8 ratioª				
< 0.40 (%)	865 (27)	79 (42.9)	944 (27.9)	ref.
0.40–0.69 (%)	894 (27.9)	46 (25)	940 (27.8)	0.49 (0.34, 0.69)
≥ 0.70 (%)	1157 (36.2)	44 (23.9)	1201 (35.5)	0.33 (0.23, 0.47)
missing (%)	708 (22.1)	21 (11.4)	729 (21.5)	–
ART use^b^	2781 (86.9)	144 (78.3)	2925 (86.4)	0.26 (0.18, 0.38)
Hypertension	626 (19.6)	82 (44.6)	708 (20.9)	2.10 (1.56, 2.84)
Diabetes	311 (9.7)	28 (15.2)	339 (10)	1.23 (0.81, 1.86)
Dyslipidemia	1466 (45.8)	101 (54.9)	1567 (46.3)	0.93 (0.69, 1.26)
Tobacco^c^	1802 (56.3)	117 (63.6)	1919 (56.7)	1.20 (0.88, 1.63)
Cocaine^c^	255 (8)	13 (7.1)	268 (7.9)	0.69 (0.40, 1.23)
Severe infections				
no severe infections	2490 (77.8)	138 (75)	2628 (77.7)	ref.
< 3 months post severe infections	45 (1.4)	10 (5.4)	55 (1.6)	5.02 (2.64, 9.56)
3–12 months post severe infections	80 (2.5)	12 (6.5)	92 (2.7)	2.32 (1.28, 4.18)
12 + months post severe infections	585 (18.3)	24 (13)	609 (18)	0.99 (0.59, 1.59)

CVD: cardiovascular disease; PY: persons-year; IQR: interquartile range; FU: follow-up; ART: antiretroviral therapy

ªWithin the last year of follow-up

^b^Defined as those who had 60 days or more of exposure to at least three antiretroviral drugs

^c^Ever use

**Fig. 2:**
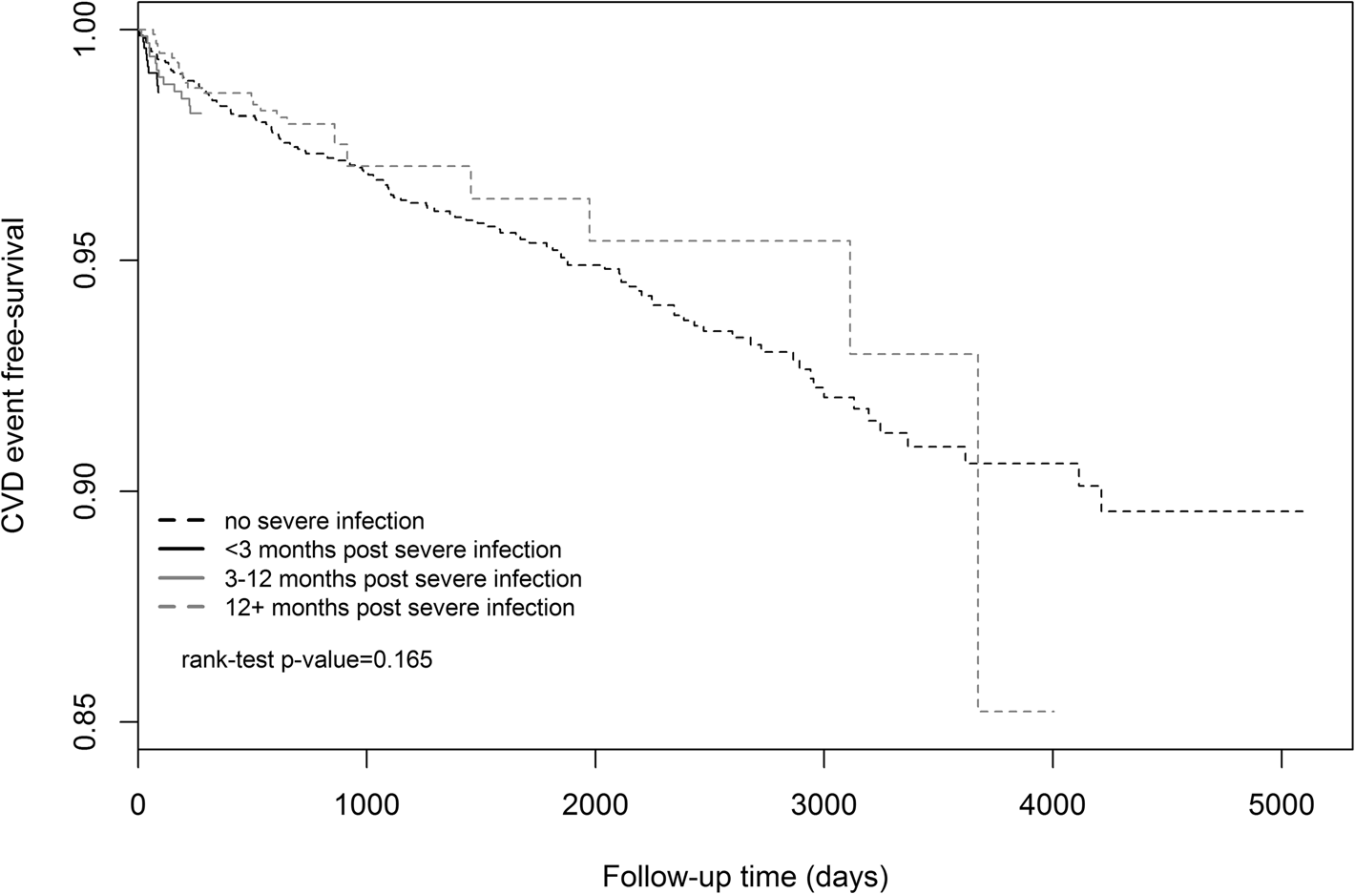
Kaplan-Meier survival curve illustrating the probability of CVD over follow-up time for patients with no severe infection and with severe infection: < 3 months post hospital discharge; 3–12 months post hospital discharge; > 12 months hospital discharge

In unadjusted analyses, non-white race/ethnicity, last HIV RNA ≥ 400 copies/mL, hypertension and tobacco use were associated with increased risk of CVD. High last CD4^+^ count (350–500 and ≥ 500 cells/mL) and high CD4^+^/CD8^+^ ratio (0.40–0.69 and ≥ 0.7) were protective against CVD (Table 1). The Kaplan-Meier survival curve (Fig. 2) illustrates the probability of developing incident CVD over time for HIV-infected individuals who never had severe infections and for those who had severe infections and developed incident CVD < 3 months, 3–12 months, and > 12 months post discharge.

**Fig. 3:**
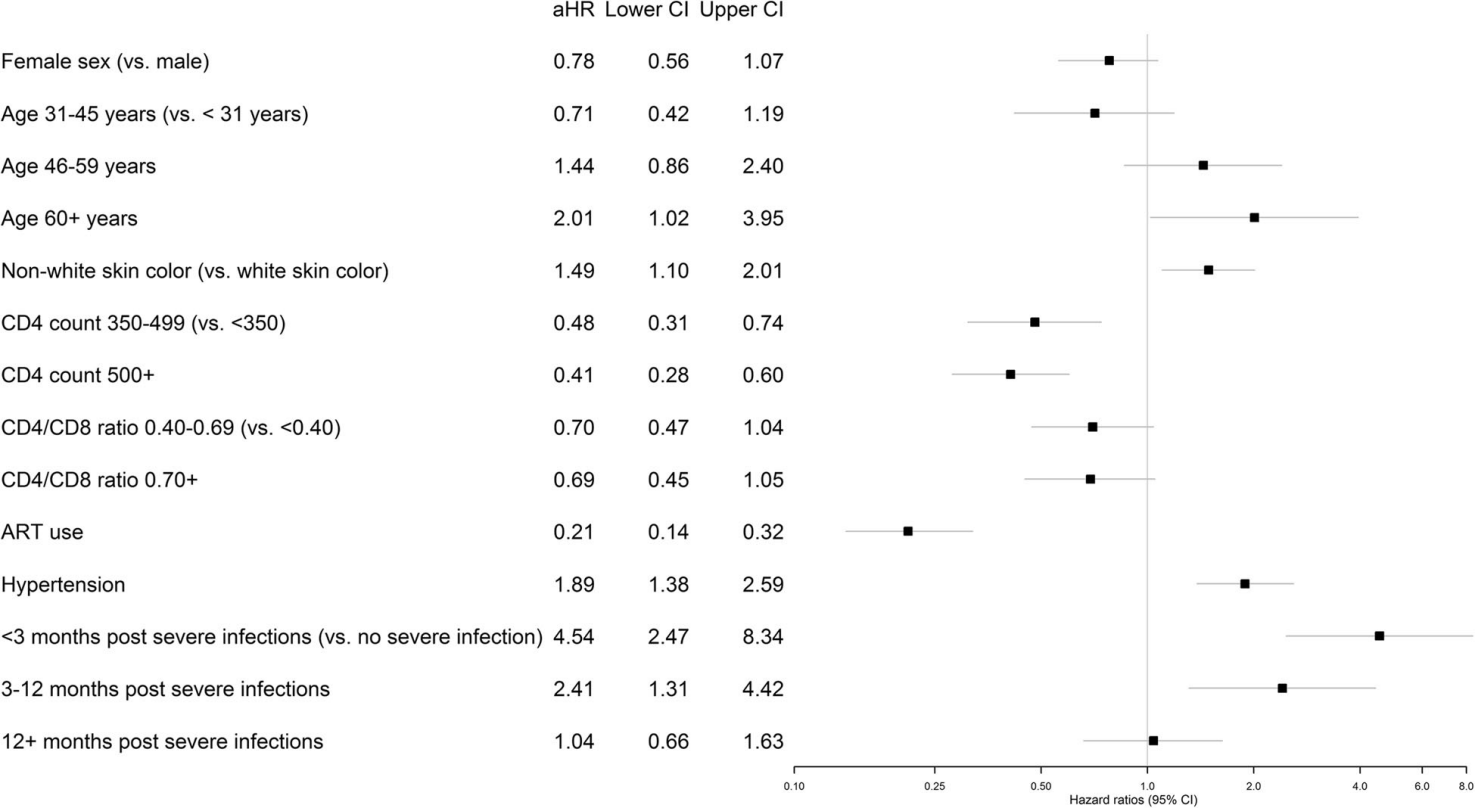
Adjusted incidence rate ratios for associations between cardiovascular disease (CVD) events and patient demographics and clinical characteristics

In the adjusted analysis, non-white race/ethnicity (adjusted hazard ratio (aHR) 1.49, *p*-value = 0.009), age ≥ 60 years (aHR 2.01, p-value = 0.045) and hypertension (aHR 1.90, p-value < 0.001) were associated with an increased hazard of CVD events, while high CD4 counts (≥ 500 cells/mm^3^: aHR: 0.41, p-value < 0.001), cART use (aHR 0.21, p-value < 0.001) reduced the hazards of CVD events. Also, high CD4+/CD8+ ratio borderline reduced the risk of CVD (≥ 0.7, aHR 0.66, p-value = 0.062) (Fig. 3).

Severe infections during follow-up was strongly associated with incident CVD (Fig. 3), greatly increasing the risk of CVD < 3 months post discharge (aHR 4.52, 95% CI 2.46–8.30) and 3–12 months post discharge (aHR 2.39, 95% CI 1.30–4.38). Similar effects were observed in the models without imputation for missing values in viral load, CD4, and, CD4+/CD8+ ratio (Additional file 3: Table S2).

## Discussion

In this study, we investigated the association between hospitalization for severe infection and subsequent risk of CVD in a large, well-characterized cohort of HIV-infected individuals in a middle-income country. We demonstrate that, after controlling for traditional and HIV-related CVD risk factors, the presence of severe infection is associated with an increase in CVD risk in the year following hospital discharge. Moreover, we observed that this association is time-dependent, higher within the first 3 months post hospital discharge (4-fold). These results is in accordance with data from the general population. For instance, Corrales-Medina and colleagues demonstrated that the association between hospital admission for pneumonia and subsequent risk of CVD is time-dependent and remains significant up to 10 years after hospital discharge [28]. In fact, hospitalization for infections is associated with an inflammatory state and a pro-coagulant activity that can persist long after infection resolves [29, 30].

Another strength of our study is the characterization of a protective effect of cART use, and high CD4 counts (> 500), on the CVD risk of HIV-infected individuals who survive hospitalization for severe infection. This data helps to build the body of evidence of an overall benefit of cART use on CVD risk [10, 31, 32]. cART is able to attenuate inflammation and endothelial dysfunction among HIV-infected individuals [32–34], factors considered to play a key role in the increased CVD risk of this population [35–41]. We have also observed a borderline association between reduced hazard of CVD events and high CD4+/CD8+ ratio (≥ 0.7), a marker of increased innate and adaptive immune activation, and higher risk of morbidity [42, 43]. This data, together with the protective effect of high CD4+ counts aforementioned, is in accordance with previous data from our group and others, demonstrating a protective effect of virologic suppression and preservation of CD4+ counts on incident CVD [8, 15].

The role of acute infection as short-term risk of CVD have been explored in the general population [28, 44–48]. Similarly, our results suggests that a past medical history of hospital admission for severe infection (at least in the past year) should be part of the clinical assessment of cardiovascular risk performed by HIV/AIDS caregivers.

There are limitations to the current study that deserve mention. Its observational design limited our ability to control for unknown sources of confounding and bias. We only captured hospitalizations at the INI hospital, which could underestimate severe infections and CVD incidences in this cohort. However, as INI is the referral center for HIV-infected individuals experiencing serious hospitalizations, missing data due to hospitalizations outside INI were likely minimized. We were not able to classify AMI into specific types [49], which could have helped to better understand the underlying mechanism of ischemia in this population. Prior data suggests a higher prevalence of type 2 AMI among HIV-infected individuals [50]. We could not fully account for the effects of chronic kidney disease as time updated glomerular filtration rates were not available. Dyslipidemia definition is dynamic and has recently been updated [51]. However, our definition is in accordance with recent publications [28, 52] and is able to capture moderate and severe cases of dyslipidemia. Moreover, dyslipidemia was not associated with CVD in our study. Important strengths of this study include the well-characterized nature of this cohort, its diversity of race and sex, and the characterization of acute CVD events beyond myocardial infarctions.

## Conclusions

We provide evidence for a time-dependent association between severe infection and incident cardiovascular disease in HIV-infected individuals from a large, clinical cohort in a middle-income country. cART use, and high CD4 count were significantly associated with reduced hazards of CVD, thus strengthening the body of evidence towards of an overall benefit of cART use on CVD risk in this population.

## Additional files

Additional file 1: **Figure S1.** Severe infections by etiology. Number of severe infections in each category, i.e. AIDS-related, bacterial, viral, fungal, or parasitic. (DOCX 25 kb)

Additional file 2: **Table S1.** Incident cardiovascular diseases among HIV/AIDS patients during follow-up. Complete list of ICD-10 codes for cardiovascular diseases and its frequency in the study. (DOCX 14 kb)

Additional file 3: **Table S2.** Unadjusted and adjusted extended time dependent Cox regression models (with no imputations for missing values in viral load, CD4 and CD4/CD8 ratio). This is the unadjusted and adjusted extended time dependent Cox regression models (with no imputations for missing values in viral load, CD4 and CD4/CD8 ratio). (DOCX 19 kb)

## Abbreviations

aHR: Adjusted hazard ratio
AIDS: Acquired Immune Deficiency Syndrome
AMI: Acute myocardial infarction
cART: Combined antiretroviral therapy
CHD: Coronary heart disease
cHR: Crude hazard ratio
CVD: Cardiovascular disease
CVDs: Cardiovascular diseases
DVT: Deep venous thrombosis
HIV: Human immunodeficiency virus
ICD-10: International Statistical Classification of Diseases and Related Health Problems
LTFU: Lost to follow-up
NCD: Non-communicable diseases
PY: Person-year
TCD4 +: CD4+ T helper cells
TCD8 +: CD8+ T cells
