# Supplementary material for: Severe infection increases cardiovascular risk among HIV-infected individuals
Source: BMC Infect Dis. 2019 Apr 11;19:319. doi: 10.1186/s12879-019-3894-6 (PMC6460818; doi:10.1186/s12879-019-3894-6)
Supplement: Supplementary file 1 — Figure S1. Severe infections by etiology. Number of severe infections in each category, i.e. AIDS-related, bacterial, viral, fungal, or parasitic. (DOCX 25 kb) [file 12879_2019_3894_MOESM1_ESM.docx]

**Severe infections during FU (n=756)**

**Bacterial (n=168)**

**Fig. S1** Severe infections by etiology

**AIDS (n=523)**

**Fungal (n=14)**

**Parasitic (n=6)**

**Viral (n=45)**
