# Supplementary material for: Severe infection increases cardiovascular risk among HIV-infected individuals
Source: BMC Infect Dis. 2019 Apr 11;19:319. doi: 10.1186/s12879-019-3894-6 (PMC6460818; doi:10.1186/s12879-019-3894-6)
Supplement: Supplementary file 2 — Table S1. Incident cardiovascular diseases among HIV/AIDS patients during follow-up. Complete list of ICD-10 codes for cardiovascular diseases and its frequency in the study. (DOCX 14 kb) [file 12879_2019_3894_MOESM2_ESM.docx]

**Table S1. Incident cardiovascular diseases among HIV/AIDS patients during follow-up.**

| **Cardiovascular disease** | **Events (%)** | **ICD-10** |
| --- | --- | --- |
| Coronary heart disease (CHD) | 76 (41.30) | I20.9, I21, I21.9, |
| Stroke | 34 (18.47) | I61.9, I62.0, I63, I63.9, I64, I63.9 |
| Peripheral arterial disease | 15 (8.15) | I74.3, I74.3 |
| Deep venous thrombosis (DVT) and pulmonary embolism | 12 (6.52) | I82.8, I82.9, I26.9 |
| Other cardiovascular diseases* | 47 (25.54) | I42, I47.1, I48, I49.0, I49.9, R57 |

*Disorders of heart muscle (cardiomyopathy) and rhythm.
