# Supplementary material for: Severe infection increases cardiovascular risk among HIV-infected individuals
Source: BMC Infect Dis. 2019 Apr 11;19:319. doi: 10.1186/s12879-019-3894-6 (PMC6460818; doi:10.1186/s12879-019-3894-6)
Supplement: Supplementary file 3 — Table S2. Unadjusted and adjusted extended time dependent Cox regression models (with no imputations for missing values in viral load, CD4 and CD4/CD8 ratio). This is the unadjusted and adjusted extended time dependent Cox regression models (with no imputations for missing values in viral load, CD4 and CD4/CD8 ratio). (DOCX 19 kb) [file 12879_2019_3894_MOESM3_ESM.docx]

**Table S2. Unadjusted and adjusted extended time dependent Cox regression models (with no imputations for missing values in viral load, CD4 and CD4/CD8 ratio).**

|  | **crude HR** | **adjusted HR** |
| --- | --- | --- |
|  | **(95% CI)** | **(95% CI)** |
| Sex |  |  |
| Male | ref. | ref. |
| Female | 0.80 (0.58, 1.10) | 0.79 (0.57, 1.09) |
| Age at end of FU |  |  |
| ≤30 years | ref. | ref. |
| 31-45 years | 0.53 (0.31, 0.89) | 0.72 (0.42, 1.22) |
| 46-59 years | 0.97 (0.58, 1.61) | 1.40 (0.82, 2.38) |
| ≥60 years | 1.34 (0.69, 2.60) | 1.95 (0.98, 3.90) |
| Race/ethnicity |  |  |
| white | ref. | ref. |
| non-white | 1.56 (1.16, 2.09) | 1.46 (1.07, 1.97) |
| Educational level |  |  |
| up to 9 years | ref. |  |
| more than 9 years | 0.67 (0.50, 0.90) |  |
| Last HIV RNA (copies/mL) | |  |
| < 400 | ref. |  |
| ≥400 | 2.41 (1.75, 3.31) |  |
| Missing | 0.81 (0.46, 1.42) |  |
| Last CD4 (cells/mm³) |  |  |
| <350 | ref. | ref. |
| 350-499 | 0.40 (0.26, 0.62) | 0.47 (0.30, 0.73) |
| ≥500 | 0.27 (0.19, 0.37) | 0.40 (0.26, 0.62) |
| Missing | 0.13 (0.05, 0.34) | 0.07 (0.02, 0.20) |
| Last CD4:CD8 ratio |  |  |
| <0.40 | ref. | ref. |
| 0.40-0.69 | 0.44 (0.30, 0.63) | 0.74 (0.48, 1.14) |
| ≥0.70 | 0.29 (0.20, 0.42) | 0.63 (0.39, 1.03) |
| Missing | 0.42 (0.24, 0.74) | 2.07 (1.02, 4.19) |
| ART use^a^ | 0.26 (0.18, 0.38) | 0.19 (0.13, 0.29) |
| Hypertension | 2.10 (1.56, 2.84) | 1.92 (1.39, 2.64) |
| Diabetes | 1.23 (0.81, 1.86) |  |
| Dyslipidemia | 0.93 (0.69, 1.26) |  |
| Tabacco^b^ | 1.39 (1.02, 1.87) |  |
| Cocaine^b^ | 0.69 (0.40, 1.23) |  |
| Severe infections |  |  |
| no severe infections | ref. | ref. |
| < 3 months post severe infections | 5.02 (2.64, 9.56) | 4.47 (2.45, 8.15) |
| 3-12 months post severe infections | 2.32 (1.28, 4.18) | 2.41 (1.31, 4.42) |
| 12 + months post severe infections | 0.99 (0.59, 1.59) | 1.05 (0.66, 1.66) |

CVD: cardiovascular disease; FU: follow-up; ART: antiretroviral therapy

^a^Defined as those who had 60 days or more of exposure to at least three antiretroviral drugs.

^b^Ever use.
